# Enhanced light extraction efficiency of Eu-related emission from a nano-patterned GaN layer grown by MOCVD

**DOI:** 10.1038/s41598-019-40971-2

**Published:** 2019-03-12

**Authors:** A. Lesage, D. Timmerman, T. Inaba, T. Gregorkiewicz, Y. Fujiwara

**Affiliations:** 1 Van der Waals-Zeeman Institute, University of Amsterdam, Science Park 904, 1098 XH Amsterdam, The Netherlands; 2 Division of Materials and Manufacturing Science, Graduate School of Engineering, Osaka University, 2-1 Yamadaoka, Suita, Osaka, 565-0871 Japan

## Abstract

Eu-doped GaN is a promising material for the active layer in red light emitting diodes. Although the output power of LEDs based on GaN:Eu has been increasing by a combination of structural and growth optimizations, there is still a significant limitation resulting from a poor light extraction efficiency, typical for high refractive index materials. Here we studied nanostructuring of the top of the optical active layer by nano-cubes for enhancement of the light extraction efficiency, and its effect on the optical emission characteristics. By etching nano-cubes into the active layer, we observed an increase in directional light output power of Eu^3+^ ions of up to 60%, as well as a grating effect. Simultaneously, the absorption of excitation light into the optical active layer was improved, leading to a 12.8 times increase of output power per available Eu^3+^ ion.

## Introduction

GaN-based efficient red light emitters are a key development to complement the already available blue and green colors, and realize monolithically produced full-color displays. The recently introduced Rec. 2020 standard defines a wide color gamut for ultra-high definition televisions (UHDTV)^1^. The RGB primaries used by the Rec. 2020 standard should be monochromatic light sources. Although monochromatic laser sources can be serve for the realization hereof, they are too expensive and have the speckle problem^2^. InGaN multi-quantum well structures are being used for the active component of commercially available LEDs^3–5^. However, it is difficult to achieve narrow linewidth emission from InGaN, because of the band-to-band nature of the optical transition responsible for light emission in these materials. Yet additional broadening results from the compositional fluctuation of group III elements in InGaN caused by segregation^6,7^, which practically preclude realization of red LEDs from InGaN. Doping with rare earth ions offers an alternative solution, and in particular Eu-doped GaN is a promising material to resolve the above mentioned limitations for red emitters. In the past, Eu^3+^ ions have been widely used as phosphors, for their strong red luminescence. This arises due to relatively efficient, well defined, and temperature stable transitions within the intra-4f shell, in the wavelength range around 620 nm. Previously, we have already demonstrated successful realization of GaN:Eu LEDs. Optimization of growth conditions, layer configuration and device structure have resulted in a steady increase of the light output of red emitting GaN:Eu devices over the last few years^8,9^. One important limitation of GaN-based LEDs is the light extraction efficiency (LEE), due to the large mismatch of the refractive index of the GaN structure with the surrounding medium. Different approaches have been used to counter this deficiency, like nanostructuring of the surface^10^, coupling to nanotubes^11^, or to localized surface plasmons^12^.

In this work we study the influence of surface patterning the optical active layer in GaN:Eu on the emission characteristics. We prepared three different samples, where one has a plain surface, serving as a reference, and the other two have different patterning geometries. The pattern consists of cubes organized in a square lattice and was formed by a nano-imprint lithography process. This particular choice of nano-patterning has been used, as dielectric structures of these dimensions are well-known to show nano-resonator behavior in the visible wavelength range. Such an effect could enhance the local optical density of states, and thus enhance the emission from the Eu^3+^ ions. We have studied the directional emission of these structures and observed an increase of the light output of 60% for the structured samples as compared to the reference. From additional measurements of total luminescence and time-resolved photoluminescence studies at different temperatures, we deduced that the internal quantum efficiency (IQE), defined by the ratio between the integrated area under an PL decay transient and that of a single-exponential decay with the time constant determined at low temperature (which we assume to be purely radiative), of the layers goes down at room temperature. From the temperature dependence of PL intensity, we conclude that the non-radiative recombination channels are thermally activated, with an energy of 22 meV. Finally, the expected nano-resonator effect that could enhance the radiative transitions in the Eu ions, was not observed. This is most likely a result of the non-optimized structure of the surface patterning.

## Sample Preparation

**Figure 1:**
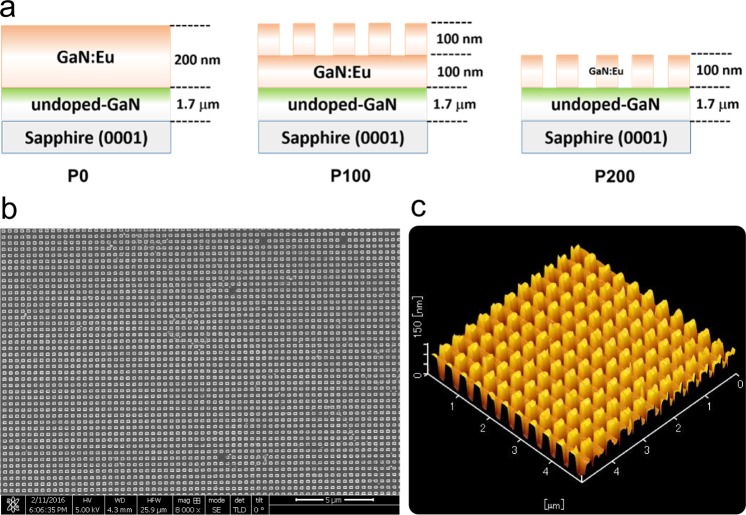
Schematics and structural characterization of the 3 samples. (**a**) Schematic cross-sections of the sample structures. (**b**) SEM and (**c**) AFM images of sample P100 (Note that the wall-structures between the cubes are a scanning artifact).

All samples have been prepared by the following growth procedure: Europium and oxygen co-doped GaN (GaN:Eu,O) was grown on double-side polished (0001) sapphire substrates by OMVPE at 100 kPa. The sample structure consisted of a 1.7 um-thick un-doped GaN layer and a 200 nm-thick GaN:Eu,O layer. Trimethylgallium, and ammonia were used as starting sources. Eu was doped using bis(n-propyl-tetramethylcyclopentadienyl)-europium [(EuCppm2)]. The resulting doping concentration of Eu^3+^ ions was 4 × 10^19^ cm^−3^. Ar-diluted O_2_ was also used for oxygen co-doping, to enhance Eu emission intensity^13^. After the growth of the GaN:Eu,O structure, resist patterns were fabricated on the samples by a standard nanoimprint lithography process. Subsequently, the samples were etched with Cl_2_ in an induced coupled plasma (ICP) etcher using the nanoimprint lithography pattern as a mask. Two samples were produced with different etching times, which are designated P100 and P200, featuring the depth of etching of 100 and 200 nm, respectively. In the final step, the resist was removed by toluene. The unpatterned layer which has been used as a reference is designated P0. A second unpatterned reference sample has also been exposed to the etching treatment in order to explore the effects of etching itself on emission characteristics, without the effect of the nano-structure. The pattern consists of cubes organized in a square lattice. The cube sides are 200 nm, height 100 nm and the edge-to-edge distance between neighboring cubes is 200 nm. Figure 1 depicts schematics of the 3 different sample designs, and a scanning electron micrograph (SEM) and atomic force microscope (AFM) image of sample P100.

## Experimental Results

**Figure 2:**
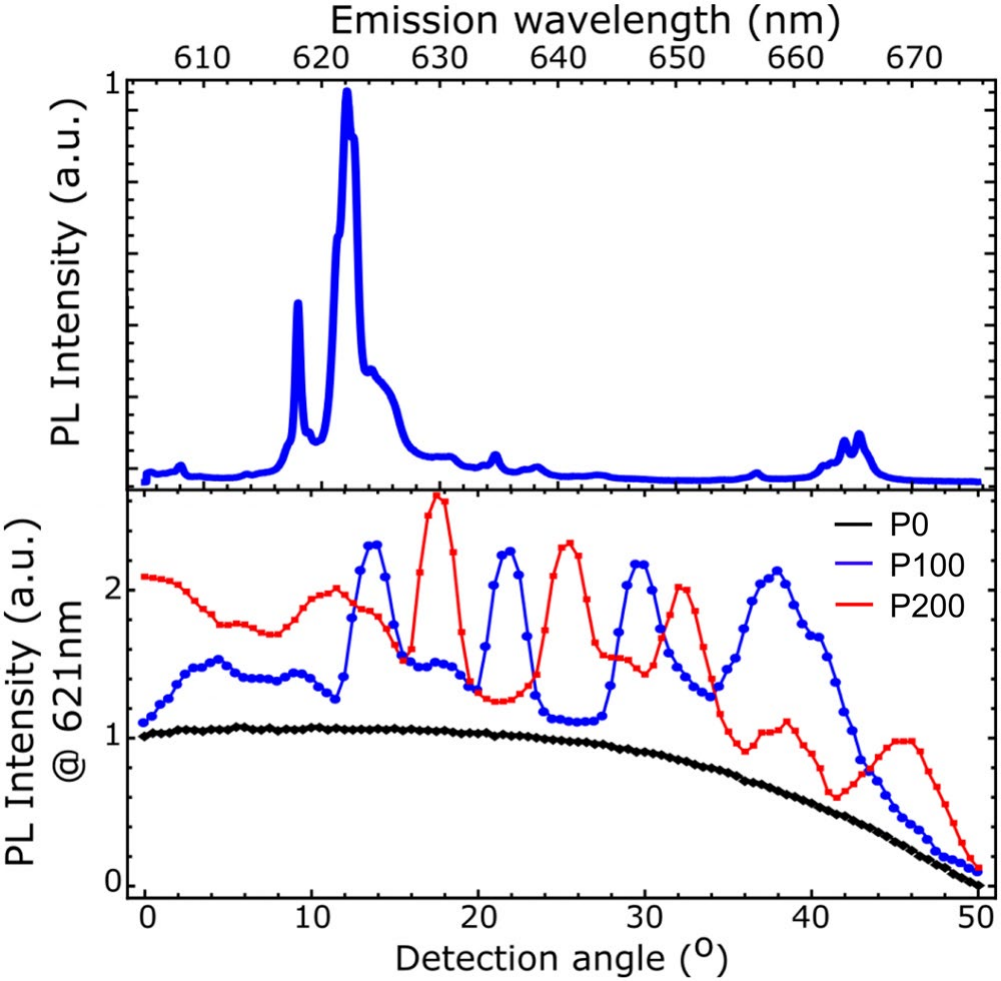
Top The RT PL spectrum showing the Eu emission lines: ^5^D_0_ to ^7^F_2_ (~621 nm) and the ^5^D_0_ to ^7^F_3_ (~665 nm). Bottom Angular dependence of the PL intensity at a detection wavelength of 621 nm.

The angular emission distribution of Eu-related luminescence for the samples was characterized in the angle-resolved PL setup under excitation of 355 nm. The angle is defined relative to the normal of the surface, results hereof are depicted in Fig. 2. The emission intensity distribution for P0 follows a flat profile, with an intensity dropping with increasing angle, thus featuring the typical escape cone for light from this sample.

For the patterned samples P100 and P200, the angular distribution looks very different, featuring peaks and dips of intensity as a function of angle. The peaks are a result of the grating-like structure of the nano-pattern, giving rise to diffracted wave guide modes, and leading to an interference effect that can be observed in the far-field^14^. In addition, it has been observed that the integrated light output perpendicular to the surface is larger for both nano-patterned samples than that of the reference. The enhancement in directional output power was 50% for P100 and 60% for P200 (see Table 1). The angular dependence of the complete emission spectrum for all three samples is shown in the Supplementary Information Fig. S2.

**Table 1:** Determined parameters for the three samples.

Sample	IQE at room temperature	Relative total output power	Relative output power in light cone	Relative volume optical active layer	Output increase normalized to Eu^3+^ ions
P0	0.34	1	1	1	1
P100	0.36	0.66	1.5	0.612	2.45
P200	0.27	0.61	1.6	0.125	12.8

Room-temperature internal quantum efficiency, relative optical output in the emission cone normal to the surface, relative volume of the optical active layer, and the output increase normalized to optical active layer volume. For all relative parameters the un-etched sample was set to 1.

Next to the angle-dependent emission, also the total emission of the samples has been determined. In this case, the samples were placed in an integrating sphere, ensuring that also the light that is not emitted in the “light cone” direction is captured. The total emitted power for all three samples decreased for increased etching times; this is especially remarkable considering the increase in emitted intensity perpendicular to the surface. The results are summarized in Table 1.

For completeness, we also investigated the emission at shorter wavelengths, in the range of the commonly appearing defect-related “yellow band” of GaN, since we observed that the intensity of this band was increased in the patterned samples. The reference layer, which received the same ICP etching treatment as P100, had a similar intensity of the “yellow band” as the unpatterned sample.

**Figure 3:**
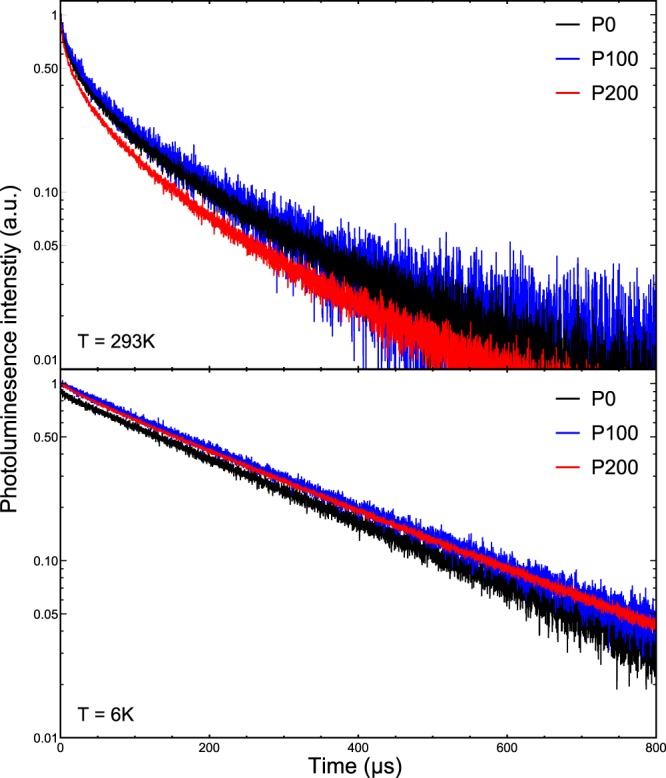
Time-dependent PL intensities at RT and 6 K.

In order to get more information about the optical activity of the Eu^3+^ ions and its possible enhancement due to nano-photonic effects, we performed a series of time-resolved PL measurements at different temperatures. The results here of are depicted in Fig. 3. At room temperature, the PL decay for all the samples has a stretched exponential characteristics. Upon temperature decrease, the dynamics for all three samples transforms towards a single exponential decay, which is reached at 10 K. This is a clear sign that non-radiative channels are thermally activated, leading to lifetime variation of individual emitters. The low temperature decay characteristics of the samples are identical in patterned and reference samples, which demonstrates that there is no alteration of the radiative rate due to the nano-patterning, but also that the IQE is likely close to unity at low temperature. As already mentioned, we have determined the activation energy of the non-radiative recombination channel to be approximately 22 meV.

## Discussion

We start by noting that, the total emitted power in all directions at RT decreases for longer etching times, resulting from a decrease in the number of available Eu ions due to the patterning of the surface. The IQE varies considerably between patterned and unpatterned samples. The temperature dependence of the IQE for all samples is depicted in Fig. S3, and the values at RT are shown in Table 1. The smaller value for the patterned samples could result from the ICP treatment, which introduces defects that might act as non-radiative recombination channels. Since a considerable amount of the optically active layer is removed by the etching process of the nanopattern, we also have to take that into account to get an estimate of the amount of Eu^3+^ ions contribution to the emission in the excited volume. Following ref.^15^ we take the penetration depth for GaN excited with a wavelength of 355 nm to be ~100 nm. The geometrical volume of the Eu-doped layer from Fig. 1a, can be estimated from the layer thickness, and the knowledge that 75% percent of the surface area is etched down. The relative volumes of the optical active layer are depicted in Table 1. Purely from a geometrical point of view of where the light can be absorbed, there is a large discrepancy with the total output power, showing an increase per Eu^3+^ ion of up to 12.8 times for P200 (Table 1).

There are three effects important here that have not been taken into account that can enhance the optical output of the etched samples. Firstly, the nano-patterning is on the scale of the incoming wavelength and can induce efficient scattering of the normal incident light into the layer. In this way, the effective path length of the light in the optical layer is increased, leading to absorption enhancement. Secondly, the nano-pattern can also induce out-of-plane scattering, and improve the LEE. A third contributing factor is a possible change of the energy transfer rate from GaN to the Eu^3+^ ions. The light is absorbed in GaN and, subsequently, nonradiative recombination of the generated carriers can excite the Eu^3+^ ions. The effectiveness of this excitation mechanism is strongly dependent on the local crystal environment of the Eu^3+^ ions, and is influenced by defects and strain^16,17^.

We found that both total emission power as well as the IQE are modified with increased etching. While the total output is most probably influenced by the decreased number of Eu^3+^ ions in the etched layer, the IQE should not be affected. From the relation between the absorption, total emission power and IQE, we estimate the maximum variation of the energy transfer efficiency to be 10%.

We therefore conclude the two first effects to be the most likely explanations for the high directional PL output in the etched samples, while having a much smaller effective concentration of Eu^3+^ ions. It is thus the enhanced scattering of the excitation light, preferentially into the optically active layer, and consecutive enhanced scattering, out of plane, of the emitted light that are most probable behind the observed increase of Eu-related PL. Even further enhancement of this effect could be achieved in the future by optimization of the nano-patterning structures, both in shape and in size.

## Conclusions

We have fabricated GaN:Eu layers, which were nano-patterned with a square lattice array of cubes, and studied effect of this on emission characteristics by PL spectroscopy. In the light emission cone perpendicular to the surface of the optical layers, we observed an increase in directional intensity of 50% and 60% for both patterned samples as compared to the unpatterned reference. Additionally, the grating-like structure of the nano-patterning gave rise to peaks and dips in the strong angular dependence of the PL intensity.

Although the amount of Eu ions in the optical excitation volume of the patterned samples was considerably lower, and there was no enhancement of the radiative rate of the Eu^3+^ ions, we observed only a minor decrease in the total output power of Eu-related emission. The observed increase of directional emission is attributed to two effects. Firstly, the increased scattering of the incoming excitation light at the nano-patterned surface, into the GaN:Eu layer, effectively increasing the path-length that excitation light travels within the GaN:Eu layer. Secondly, the same nano-patterned surface scatters emission light out of the GaN:Eu layer, a highly desired effect in a material with high refractive index such as GaN. Especially the latter could be optimized further with an eye at direct applications in GaN:Eu light emitting devices.

## Methods

An angle-resolved PL setup was purposely built to investigate directionality of emission. The system allowed both spectral, as well as time resolved measurements. A xenon lamp (L2273 Hamamatsu) coupled to a Solar MSA130 double grating monochromator, was used as excitation source for the spectral measurements. For time-resolve measurements an optical parametric oscillator (OPO) pumped by the third harmonic of a 100 Hz Nd:YAG laser, with a pulse duration of 5 ns was used. Emission was dispersed by an M266 (Solar LS) monochromator coupled to either a silicon CCD (Horiba scientific) or a photo multiplier tube (Horiba scientific) for photon detection. The sample was placed perpendicular to the rotation plane, such that the normal to the surface has the zero degree detection angle. The excitation was fixed to the sample at an angle of 20 degrees above the plane. To investigate emission independent of directionality an integrating sphere was used (Newport, 7.5 cm diameter). With excitation source a xenon lamp (L2273 Hamamatsu) coupled to a double grating monochromator (Solar MSA130). The datasets generated during and/or analyzed during the current study are available from the corresponding author on reasonable request.

## Supplementary information

Supplementary Information
