# Supplementary material for: Enhanced light extraction efficiency of Eu-related emission from a nano-patterned GaN layer grown by MOCVD
Source: Sci Rep. 2019 Mar 12;9:4231. doi: 10.1038/s41598-019-40971-2 (PMC6414605; doi:10.1038/s41598-019-40971-2)
Supplement: Supplementary file 1 — Supplementary Information [file 41598_2019_40971_MOESM1_ESM.pdf]

Enhanced light extraction efficiency of Eu-related emission from a nano-patterned GaN layer grown by MOCVD

A. Lesage[1,*], D. Timmerman[1,2], T. Inaba[2], T. Gregorkiewicz[1,2], Y. Fujiwara[2]

Supplementary Information

Total emitted power

For the determination of the total emitted power, i.e. in all directions, the samples were put in an integrating sphere and excited directly by the light from a xenon lamp around a wavelength of 340 nm. The integrating sphere ensures a homogenous light intensity field after a few reflections of the emitted light and the unabsorbed excitation light. This was probed by guiding the light to a monochromator with detector, to spectrally resolve the light. For all three samples the excitation intensity was kept the same at approximately 1 μW. The results hereof can be seen in Fig S1. We note that the intensities of the unabsorbed excitation light increases, implying more scattering, and hence less absorption.

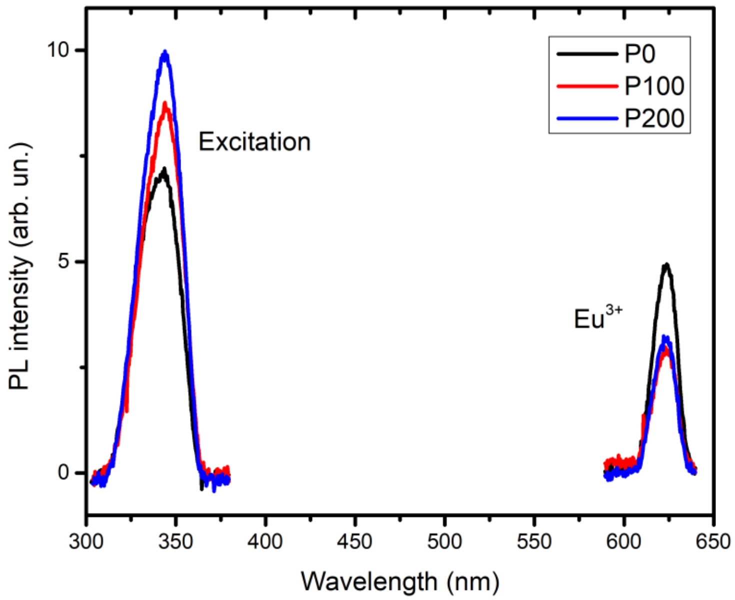

Figure S1 Integrated sphere intensities of Eu-related PL and non-absorbed excitation light.

Angle dependent full PL spectrum.

From the angle dependence of the emission spectrum shown in Fig. S2 the grating behavior becomes obvious. The tilted lines in the contours of P100 and P200 are a result of the wavelength dependent maximum angle of diffraction, which is following from the grating equation.

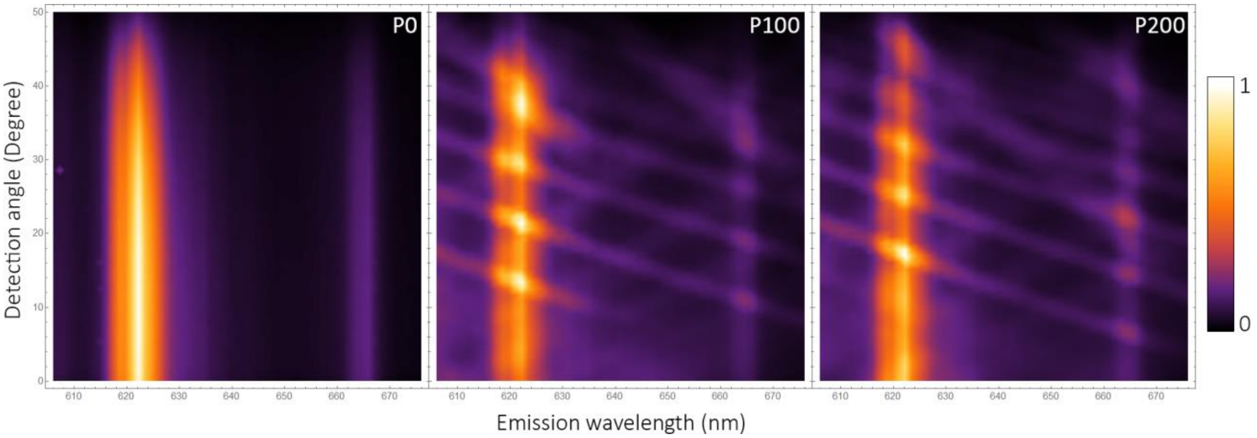

Figure S2 Normalized angle dependent spectral intensities for the three samples.

Temperature dependence internal quantum efficiency

The time-dependent PL intensity has been determined for all three samples at various temperatures. At low temperatures a nearly single-exponential decay was observed. The internal quantum efficiency of PL emission, which indicates the chance that an excited Eu^{3+} ion can emit a photon, is determined by integrating the area under the PL decay curve and dividing it by that of a single exponential decay. The results hereof are depicted in Fig. S3. At larger temperatures the IQE goes down, because thermally activated non-radiative channels become active.

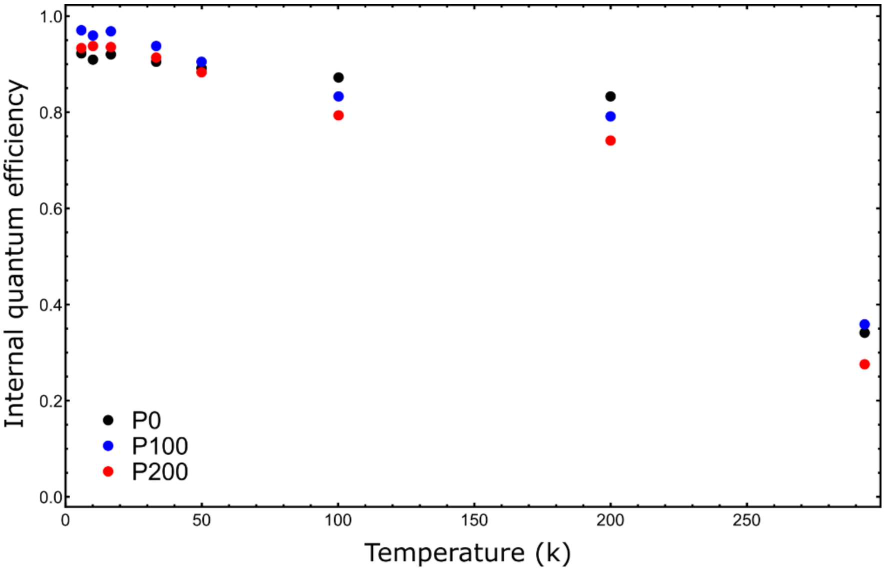

Figure S3 Temperature dependence of IQE determined from the PL lifetime measurements.

Low Temperature emission spectrum

When lowering temperature we observe narrower and stronger Eu emission lines, in agreement with the enhanced IQE. These changes in emission spectrum are as observed in literature [1].

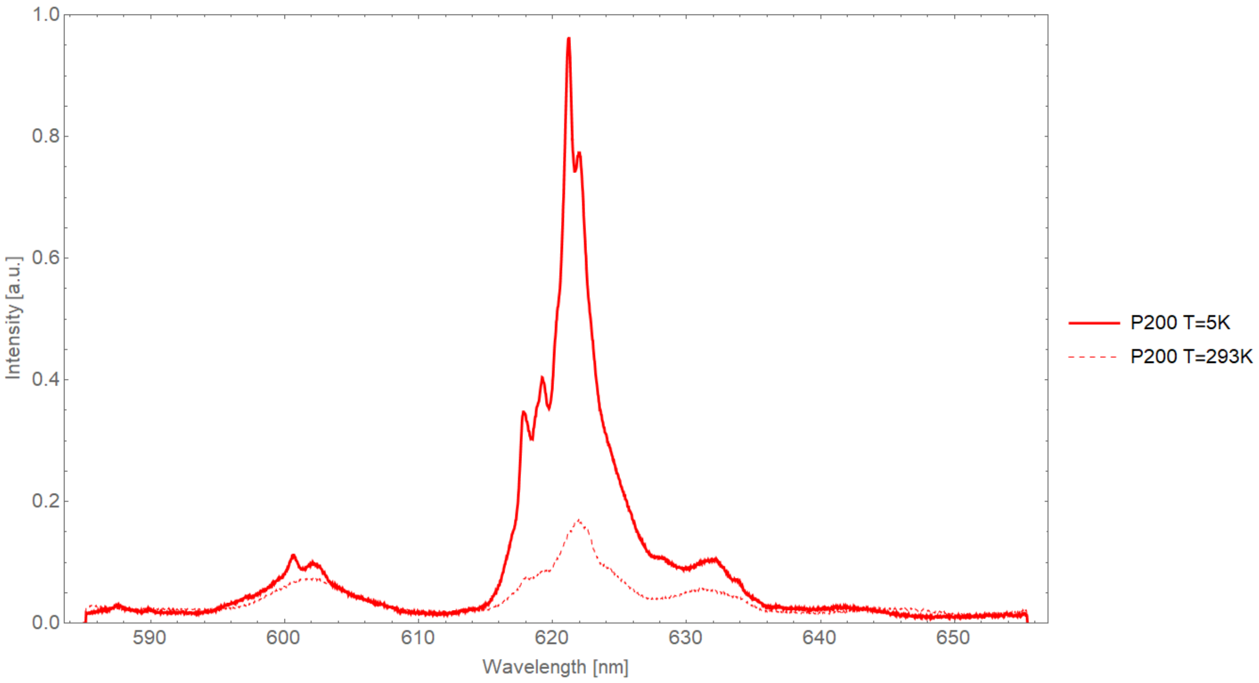

Figure S4 Photoluminescence Spectra of GaN:Eu P200 sample at RT and 6K.

[1] Nishikawa, A., et al. "Improved luminescence properties of Eu-doped GaN light-emitting diodes grown by atmospheric-pressure organometallic vapor phase epitaxy." Applied Physics Letters 97.5 (2010): 051113.
